# Aerosol interplay with meteorology in a biodiversity-rich high-altitude site in India: impacts of southwest monsoon on aerosol chemical characteristics

**DOI:** 10.1038/s41598-026-57287-7

**Published:** 2026-06-16

**Authors:** Kavyashree N. Kalkura, Aishwarya Singh, Ramesh Chand K. A., M. Devaprasad, Govindan Pandithurai, Rizana Salim, Samara Carbone, James Allan, Gordon McFiggans, Pengfei Liu, Hugh Coe, Ravikrishna Raghunathan, Sachin S. Gunthe

**Affiliations:** 1 EE Division, Department of Civil Engineering, Indian Institute of Technology Madras, Chennai, India; 2 Center for Atmospheric and Climate Sciences, Indian Institute of Technology Madras, Chennai, India; 3 Department of Mechanical Engineering, College of Engineering Munnar, Munnar, India; 4 Institute of Agrarian Sciences, Federal University of Uberlândia, Uberlândia, MG Brazil; 5 Department of Earth and Environmental Sciences, School of Natural Sciences, University of Manchester, Manchester, UK; 6 National Centre for Atmospheric Science, University of Manchester, Manchester, UK; 7 School of Earth and Atmospheric Sciences, Georgia Institute of Technology, Atlanta, GA USA; 8 Department of Chemical Engineering, Indian Institute of Technology Madras, Chennai, India; 9 Present Address: Aerosol Chemistry Department, Max-Planck Institute for Chemistry, Mainz, Germany

**Keywords:** Aerosol Chemical Composition, Secondary Organic Aerosol (SOA), Positive Matrix Factorization (PMF), Southwest Monsoon, Western Ghats, Climate sciences, Ecology, Ecology, Environmental sciences

## Abstract

Atmospheric aerosols significantly impact climate, and the hydrological cycle, in high-altitude regions like the Western Ghats in India, where monsoon dynamics play a critical role. Here we present the first Q-ACSM-based characterization of non-refractory submicron aerosol (NR-PM_1_) and organic aerosol (OA) source apportionment from Munnar, Kerala, during the Southwest monsoon season (06 June—25 July 2021). The organics comprised the largest fraction of NR-PM_1_ (42.1 ± 23.7%), closely followed by sulfate (41.0 ± 19.6%). Aerosol composition was strongly controlled by meteorological regime: sulfate dominated during active monsoon periods and exhibited a persistent high-speed W–SW transport signature consistent with strong marine influence, while organics became the dominant fraction (52%) during the mixed period. OA source apportionment, conducted using Positive Matrix Factorization (PMF), identified four key factors: hydrocarbon-like OA (HOA, 11.4 ± 10.4%), biomass-burning OA (BBOA, 14.0 ± 13.8%), Biomass-Burning-derived Oxygenated OA (BB-OOA, 40.2 ± 23.0%), and Oxygenated OA (OOA, 34.3 ± 24.3%), suggesting dominance of secondary aerosols in the region. These findings highlight the contrasting roles of long-range marine transport and local secondary processing in shaping fine aerosol composition at high-altitude monsoon sites, with implications for aerosol hygroscopicity and cloud condensation nuclei activity over the Western Ghats.

**Supplementary Information:**

The online version contains supplementary material available at https://doi.org/10.1038/s41598-026-57287-7.

## Introduction

Submicron atmospheric aerosols are of wide concern due to their direct and indirect impacts on climate^1,2^, air-quality^3–5^, and human health^6–8^. These aerosols exhibit a large diversity in their sources, size distribution, chemical composition, and lifetimes. Crucially, they play a pivotal role in cloud formation and perturbing cloud properties by scattering solar radiation^9^ and acting as cloud condensation nuclei (CCN)^10,11^, thereby influencing the global radiative budget^12^ and impacting the hydrological cycle^13,14^. Clouds, in turn, influence the life cycles of gaseous species and particulates by providing a medium for aqueous-phase reactions and by contributing to deposition pathways^15,16^.

Organic aerosols (OA), which contribute 20–90% of submicron aerosol mass in remote regions^17^, originate from primary emissions (e.g., biomass burning^18^, fossil fuels^19,20^ and biological organisms^21,22^) or secondary formation via gas-to-particle conversion processes like condensation, nucleation, and various chemical reactions^20,23^. While primary organic aerosol (POA) concentration is governed by source emissions, dilution, and transportation, secondary organic aerosol (SOA) formation is a more complex process involving oxidants, radicals, and meteorological factors such as temperature, relative humidity, and photochemical aging^24,25^. In tropical environments, biogenic volatile organic compounds (VOCs) from vegetation drive active SOA formation, further enhanced by anthropogenic emissions^26–28^. SOA modifies aerosol hygroscopicity and optical properties, with cascading effects on CCN activity, aerosol-cloud interactions, and regional radiative forcing, particularly in monsoon systems^29,30^.

Much like the East Asian and the South Asian summer monsoon systems^31^, the Indian southwest monsoon (SWM), responsible for ~ 80% of annual rainfall in the subcontinent, is highly sensitive to aerosol perturbations^32,33^. Increased aerosol mass loading has been shown to modulate cloud microphysics, regional circulation and precipitation during the SWM, at times leading to extreme rainfall events via aerosol–cloud microphysical feedbacks^34^. Despite the well-recognized importance of aerosols in monsoon dynamics, real-time chemical characterization of submicron aerosols during the SWM period remains limited. Prior studies in India have focused on urban or semi-urban sites (e.g., Kanpur ^35,36^, Bhubaneswar^37^ , Chennai^38^) or the Western Ghats site Mahabaleshwar^39–42^, which experiences varying influences from regional anthropogenic emissions, marine air masses, and seasonal transport pathways. For example, Mukherjee et al. reported primary OA dominance during the monsoon at Mahabaleshwar, while Singla et al.^40^ identified organonitrate and organosulfate formation during pre-monsoon, highlighting the role of photochemical and nocturnal oxidation. However, no study has conducted real-time online measurements of non-refractory PM_1_ (NR-PM_1_) chemical composition or OA source apportionment during the active SWM at a high-altitude, relatively pristine site in the southern Western Ghats. Existing work either relied on offline filter-based techniques^43^ or did not resolve meteorological sub-regimes within the monsoon^40^.

The Western Ghats, a biodiversity hotspot prone to extreme rainfall and landslides^44,45^, experiences daily precipitation exceeding 300 mm during the SWM^46^. Understanding aerosol–cloud interactions in this region is critical for predicting hydrometeorological extremes, yet chemical characterization of aerosols during this season is sparse. The 2021 COVID-19 lockdown provided a unique opportunity to study aerosols under reduced anthropogenic emissions^47^. At Munnar (1600 m a.s.l.), a high-altitude hill-station, where tourism and vehicular traffic dominate local emissions, the lockdown effectively suspended these activities, enabling measurements under near-pristine conditions. This allowed clearer isolation of biogenic SOA from forest/tea plantations, marine-derived sulfate from the Arabian Sea, and biomass-burning-influenced OA.

Here, we present the first real-time characterization of NR-PM_1_ chemical composition and OA source apportionment during the active SWM at Munnar in the southern Western Ghats, using a quadrupole aerosol chemical speciation monitor (Q-ACSM). This study was conducted at the Natural Aerosol and Bioaerosol High-Altitude (NABHA) laboratory, Munnar (10.0928°N, 77.0675°E), during June-July 2021 — a period coinciding with both the active SWM and the second phase of the COVID-19 lockdown. However, the tea-factories in the area were still operational, leading to biomass burning emissions. This rare circumstance allowed the biogenic SOA signal from dense forest and tea plantation emissions, marine-derived sulfate from the Arabian Sea, and biomass-burning-influenced organic aerosol to be isolated with much greater clarity than would be possible under normal anthropogenic emission levels.

We quantify primary: hydrocarbon-like OA (HOA), biomass-burning OA (BBOA), and secondary: oxygenated OA (OOA), biomass-burning-influenced OOA (BB-OOA), contributions using positive matrix factorization (PMF) technique and examine their modulation by meteorological regimes (SW-wind-dominant vs. mixed-wind periods). By leveraging the COVID-19 lockdown as a natural experiment, we further assess how reduced anthropogenic emissions influenced SOA formation pathways. Our results provide novel insights into aerosol–cloud interactions with implications for regional precipitation processes in this climatically sensitive terrain.

## Methods

### Study site and measurement period

Online, real-time measurements were conducted from 06 June to 25 July 2021, at the NABHA Laboratory, situated in a high-altitude site in Munnar (1600 m a.s.l) in the Western Ghats of India. The sampling site is located on the premises of the College of Engineering Munnar (CEM), which, being a rural site, is surrounded by tea gardens, residential areas, lodging facilities and reserve forests. The laboratory is located ~ 90 km inland from the Arabian Sea and experiences heavy rainfall from June to September. The site is accessible by a road that leads uphill from the Munnar town. The region is influenced primarily by biogenic vegetation and marine sources, as the air masses originate from the ocean and pass through the tea gardens and lush green forests of the Western Ghats.

### Instrumentation

#### Aerosol chemical speciation monitor

Concentrations of non-refractory submicron aerosol (NR-PM_1_) species, including organics, Sulfate (SO_4_^2-^), Ammonium (NH_4_^+^), Nitrate (NO_3_^-^) and Chloride (Cl^-^) were measured using the Quadrupole Aerosol Chemical Speciation Monitor (Q-ACSM, Aerodyne Research Inc.). Ambient aerosols were sampled through an inlet mounted on the laboratory roof and distributed to the instruments via a manifold in a temperature-controlled room (~ 24 °C). To mitigate the effects of high ambient humidity during the monsoon season, the sample air was dried (RH < 40%) using silica gel driers prior to analysis by the instruments. Within the Q-ACSM, the sample air is drawn at 0.085 lpm, where NR-PM_1_ particles are flash-vaporized on a tungsten filament maintained at ~ 600 °C and subsequently ionized by 70 eV electron impact. Post ionization, the chemical species are analyzed by a quadrupole mass spectrometer and recorded at a 15-min time resolution.

Flow calibration was performed to determine the instrument’s volumetric flow rate. The particle lens pressure was recorded over a range of flow rates, measured independently using a Gilibrator (bubble flow meter). A linear regression of this relationship was used to derive the slope and intercept for the volumetric flow calculation. Mass calibration was carried out using ammonium nitrate and ammonium sulfate as chemical standards. Monodisperse 300 nm particles were generated with a nebulizer and size-selected using an electrostatic classifier (TSI 3080), with number concentrations quantified by a condensation particle counter (TSI 3776)^48^.

The Q-ACSM data was processed using the ACSM Local Software (ACSM_Local_v1.60.3), provided by ARI, written in IGOR Pro software (version 6.37, Wave metrics), along with the fragmentation data provided by Allan et al.^49^ Based on the mass calibration, relative ionization efficiencies (RIEs) of 5.62 for ammonium and 1.20 for sulfate were used. Default RIE values were used for organics (1.4), chloride (1.3), and nitrate (1.3). A collection efficiency of 0.5 was applied to the dataset to account for particle bounce at the standard vaporizer, a standard practice for ambient aerosol measurements^50–52^. Detailed QA/QC procedures, calibration uncertainties, and detection limits for the Q-ACSM are provided in Sect. 1.2 of the Supplementary Information.

#### Black carbon measurement

Black carbon (BC) mass concentrations were measured using a seven-wavelength AE-33 Aethalometer (Magee Scientific), operating at wavelengths of 370, 470, 520, 590, 660, 880, and 950 nm at a 1-min time resolution. BC mass concentrations were derived from light attenuation at 880 nm using a mass absorption cross-section (MAC) of 7.77 mg^-153^. The AE-33 incorporates a dual-spot compensation system for real-time correction of the filter-loading artifact^53^. The instrument sampled from the same inlet manifold as the Q-ACSM. BC data were averaged to a 15-min time base for consistency with ACSM measurements in all cross-species analyses.

#### Meteorological measurements

Key meteorological parameters, including ambient temperature (ºC), relative humidity (%), wind direction (º) and speed (m s^-1^) were recorded using a Thies CLIMA automatic weather station (AWS). However, the AWS dataset was not available during the initial period of the campaign (06 to 12 June 2021). Precipitation was not measured at our study location; however, the rainfall measured using a tipping-bucket rain gauge (Campbell Scientific TB4) operated by the National Centre for Earth Science Studies (NCESS) at the Cloud Physics Observatory in Munnar, located approximately 10 km from the study site, was used (Figure S2).

### Data analysis

#### Positive matrix factorization (PMF) analysis

Source apportionment of OA mass spectra was performed using the positive matrix factorization (PMF) model. The organic mass concentration and error matrices were exported from the ACSM software and then analyzed using the PMF Evaluation Tool (PET, version 2.06)^54^, in Igor Pro. The analysis and factor evaluation followed the procedures outlined by Zhang et al. and Ulbrich et al.^54,55^. In this study, m/z from 12 to 100 were included, as most organic compounds are covered in this range. The PMF model mathematically decomposes the input data matrix (X) into two matrices: factor contributions (G, time series) and factor profiles (F, mass spectra), leaving a residual matrix (E) (Eq. 1).

1 *X = GF + E*

In this study, we have performed source apportionment analysis for the entire campaign period. The final factor profiles were determined based on a careful examination of *Q/Q*_*exp*_ values (Table S1 and Fig. S1), residuals, and the correlation of PMF factors with external tracers and the reference mass spectra. The correlation values of the PMF mass spectra and the reference mass spectra are given in Table S2.

#### Nonparametric wind regression analysis

To investigate the potential geographical source regions of the measured aerosol species and PMF factors, a Nonparametric Wind Regression (NWR) analysis was performed. This analysis combines ambient concentration data with real time wind speed and direction measurements^56,57^. The predicted concentration at a particular wind direction and speed (θ, *u*) are calculated using the non-parametric regression formulas as described in Yu et al. ^58^. The main objective of this method is to estimate the concentration near to the concentration at θ and *u* using weighted average and gaussian kernel function^59^. We used the Zefir software package developed by Petit et al.^59^, to carry out the wind analysis and calculate the concentration simultaneously generating plots from the time-averaged ACSM and PMF factor data. The plots were generated for an angle resolution of 1° and a radial resolution of 0.25 m s^-1^.

## Results and discussions

### Temporal variation of NR-PM_1_ chemical composition and meteorological parameters

**Fig. 1:**
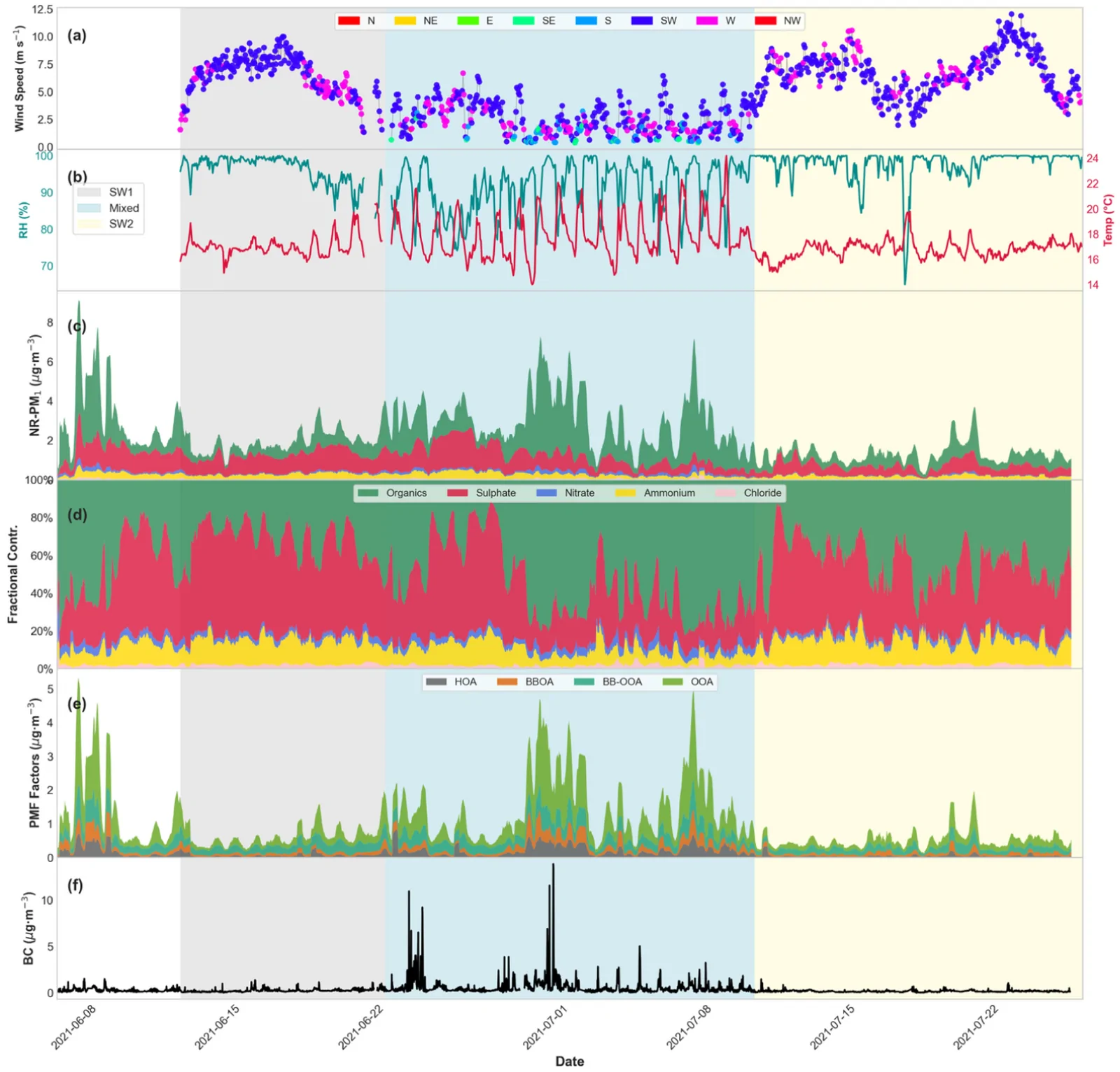
Time series of meteorological parameters and the chemical composition of submicron aerosols (PM_1_) from 06 June to 25 July, 2021. The background shading delineates three distinct periods based on prevailing air mass influences: SW1 (southwesterly winds; grey), Mixed (light blue), and SW2 (southwesterly winds; light yellow). **(a**) Wind speed (in m s^-1^), color-coded with wind direction (in º) (**b**) Ambient relative humidity (RH, in %) shown on the left y-axis and temperature (Temp, in °C) on the right y-axis (**c**) Absolute mass concentrations (in µg m^-3^) of the major non-refractory PM₁ (NR-PM₁) chemical species: organics, sulfate, nitrate, ammonium, and chloride. (**d**) The fractional contribution (%) of each chemical species to the total NR-PM₁ mass. (**e**) Mass concentrations of organic aerosol (OA) factors identified via Positive Matrix Factorization (PMF) analysis. The factors include hydrocarbon-like OA (HOA), biomass burning OA (BBOA), oxygenated OA (OOA), and BB oxygenated OA (BB OOA). (**f**) Time series of the mass concentration (in µg m^-3^) of black carbon (BC).

Figure 1 provides a comprehensive overview of the temporal evolution of key meteorological parameters and the chemical characteristics of non-refractory submicron aerosol (NR-PM_1_) at the high-altitude site during the southwest monsoon campaign. The data reveals that aerosol concentration and composition are strongly modulated by changes in wind direction and precipitation intensity. Accordingly, the sampling period was classified into three distinct phases: a Southwest Wind Dominant Period 1 (SW1) from 12 to 21 June where the wind was predominantly from the southwest; a Mixed Period (Mixed) from 22 June to 09 July, characterized by lower wind speeds and variable wind directions from the east, south, and west; and again a Southwest Wind Dominant Period 2 (SW2) from 10 to 25 July, (Fig. 1). The SW1 and SW2 periods correspond to the active monsoon spells, while the mixed periods can be considered as a break between these heavy precipitation spells.

The prevailing winds were predominantly from the west and southwest directions, with a campaign-average speed of 4.9 ± 2.8 m s^-1^. Wind speeds were significantly higher during SW1 (6.45 ± 1.81 m s^-1^) and SW2 (6.79 ± 1.97 m s^-1^) compared to the Mixed period (2.44 ± 1.42 m s^-1^; p < 0.001 for both pairwise comparisons, Table S3). The campaign-average temperature was 17.3 ± 1.5 ºC and relative humidity was 94.6 ± 6.4%. The Mixed period was characterized by significantly higher temperature (17.92 ± 1.76 °C vs. 17.07 ± 0.86 °C in SW1, p < 0.001) and significantly lower relative humidity (91.39 ± 6.90% vs. 96.17 ± 3.91% in SW1, p < 0.001), consistent with reduced marine air mass influence and weaker wet scavenging during this period. Precipitation was higher during SW1 and SW2 (Figure S2), while the Mixed period experienced drier conditions, as confirmed statistically (Table S3).

The average NR-PM_1_ concentration over the entire measurement period was 2.28 ± 1.81 µg m^-3^ (mean ± 1 s.d.), in which organics comprised a significant fraction (1.15 ± 1.49 µg m^-3^, 42.1%), followed by sulfate (0.79 ± 0.48 µg m^-3^, 41%), ammonium (0.22 ± 0.21 µg m^-3^, 11.2%), nitrate (0.08 ± 0.09 µg m^-3^, 3.5%) and chloride (0.04 ± 0.06 µg m^-3^, 2.2%) (Fig. 1c, d). Such low average concentrations suggest that the site is relatively free of persistent urban pollution, though it exhibits high temporal variability. In comparison, while the average concentration of organics in the present study was higher than that observed in the long-term study conducted at Jungfraujoch (an alpine site at 3580 m a.s.l., in Switzerland, Europe),^60^ the concentrations of the other species remained similar to those reported there.

**Fig. 2:**
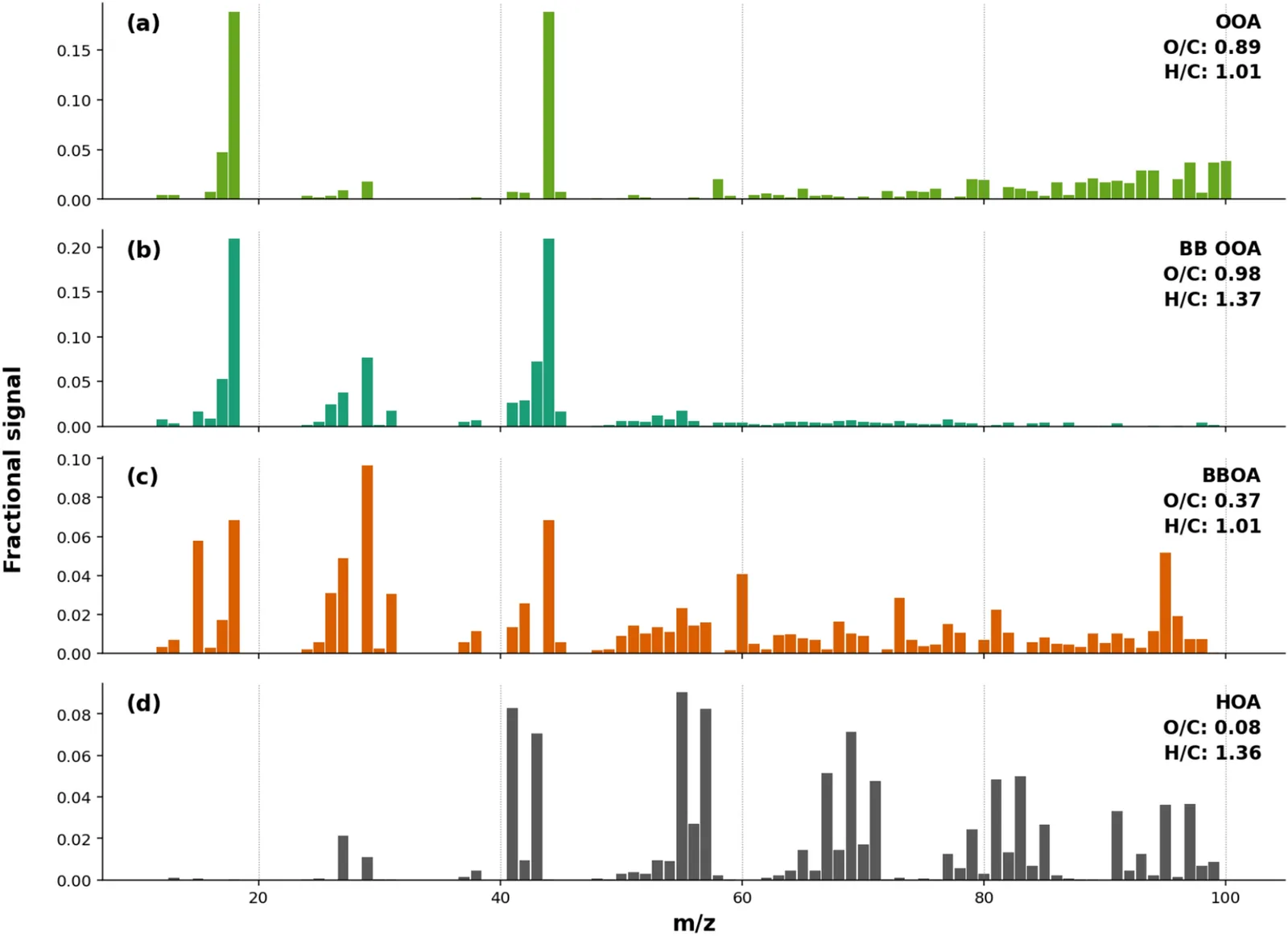
Mass spectral profiles for the four-factor PMF solution result from the OA measured during the Munnar campaign, comprising two primary (HOA and BBOA) and two secondary (OOA and BB-OOA) factors.

The source apportionment of the OA yielded a four-factor solution with two primary factors: Biomass-burning OA (BBOA) and Hydrocarbon-like OA (HOA), and two secondary factors: Biomass-burning Oxygenated OA (BB-OOA) and highly aged Oxygenated OA (OOA). The factor profile of these factors is shown in Fig. 2. The individual contributions of PMF factors were HOA 0.13 ± 0.25 µg m^-3^ (11.4 ± 10.4%), BBOA 0.13 ± 0.22 µg m^-3^ (14.0 ± 13.8%), OOA 0.28 ± 0.23 µg m^-3^ (34.3 ± 24.3%), and BB-OOA 0.47 ± 0.66 µg m^-3^ (40.2 ± 23.0%) (Fig. 1e). Overall, OOA and BB-OOA together contributed 74.5%, indicating that organic aerosols are highly oxidized in the study region, which is similar to the reported fraction of OOA over the high-altitude site of Jungfraujoch^60^. Among Indian high-altitude sites, Mukherjee et al. reported a campaign-average NR-PM₁ of 7.5 ± 6.5 µg m⁻^3^ at Mahabaleshwar^39^ (1348 m a.s.l., Western Ghats), approximately 3.3 times higher than the present study, with organics (59%) and sulfate (23%) as the dominant species. During the monsoon season specifically, only one oxygenated OA factor was resolved, contributing ~ 20% to total OA, in stark contrast to the secondary-OA-dominated composition (74.5% OOA + BB-OOA) observed here^40^. During the low concentration monsoon periods, this site was also sulfate dominated^41^. The lower absolute concentrations at our Munnar site reflect its more pristine southerly location, farther from continental source regions, and the strong dilution and wet scavenging associated with the active SW monsoon.

**Fig. 3:**
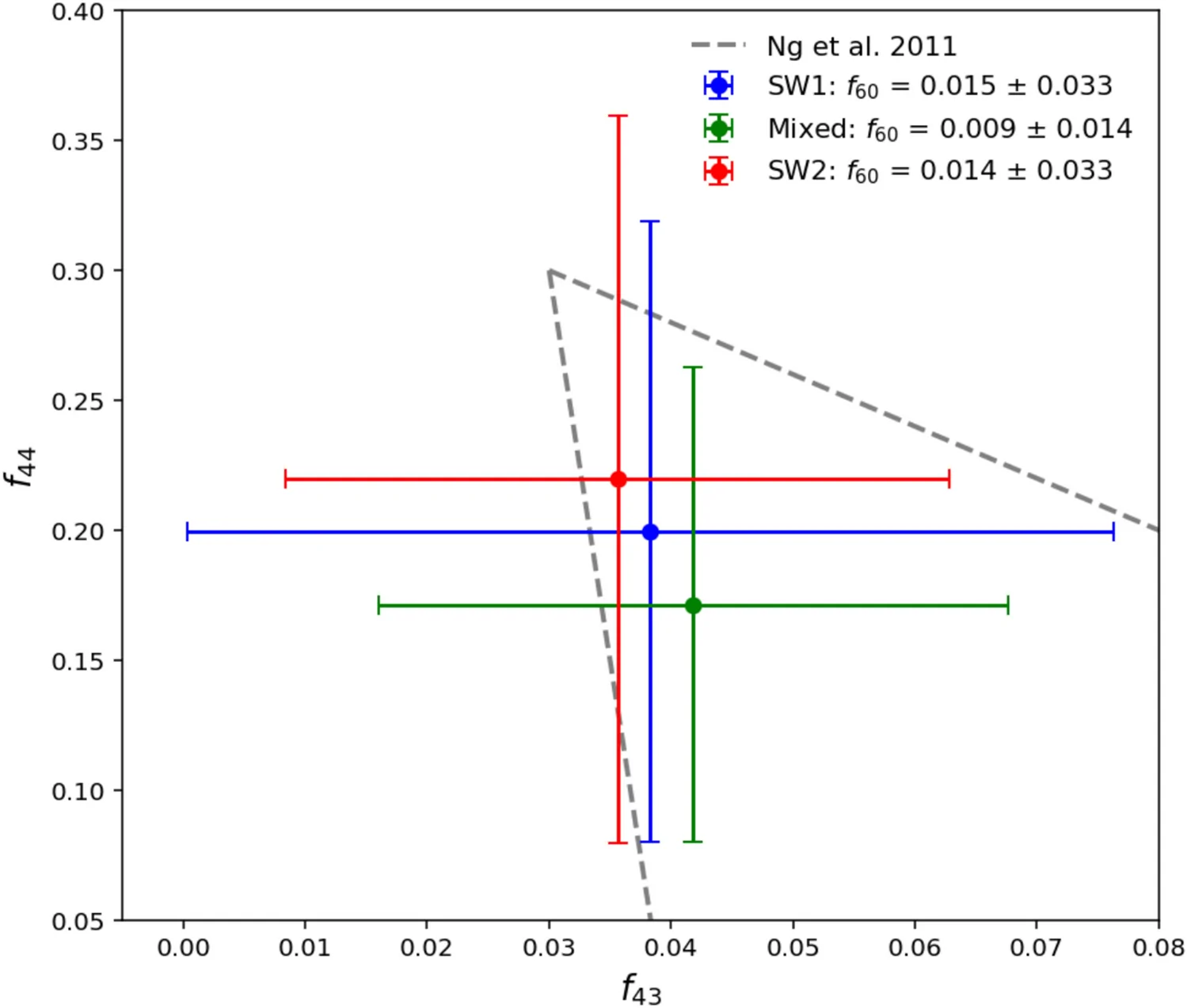
The average *f*_44_ vs. *f*_43_ values for different sampling periods classified by meteorological conditions (SW1, Mixed, and SW2). The error bars indicate one standard deviation. The data are shown in the context of the reference triangle for ambient aerosol from Ng et al., 2011^67^ (gray dashed lines).

The black carbon (BC) concentration also showed distinct variation during the sampling period, with a campaign-average value of 0.36 ± 0.65 µg m^-3^ (Fig. 1f). The concentration increased during the mixed period, highlighting the dominance of combustion sources in the vicinity of the sampling location. OA also increased during the same time period (Figs. 1c and 1d). As wind speeds were low during the mixed period, BC and OA loadings are most likely of local origin, as indicated by their higher *f43* values (Fig. 3), consistent with a relatively less-oxidized, fresher organic aerosol composition compared to SW periods, as depicted by relatively higher POA in Figure S3.

BC exhibited weak correlations with all PMF-resolved organic aerosol factors and ACSM species throughout the campaign. The highest correlation was observed with biomass-burning-influenced oxygenated organic aerosol (BB-OOA), although the correlation coefficients remained low (R^2^ = 0.08–0.14) (Figure S4). This weak co-variability reflects the strong influence of meteorology and wet scavenging on BC at our site, particularly during the SWM periods^61^. The relatively higher correlation with BB-OOA likely reflects co-emission of BC and biomass-burning-related organic aerosol from nearby combustion sources, followed by rapid oxidative processing of organic vapors under high relative humidity conditions^62,63^. Such rapid aging can lead to the formation of BB-OOA on short timescales, resulting in modest linear correlations despite a shared source influence. Biomass-fueled tea-processing activities from 16 tea-factories in the vicinity of the site likely represent an important local source of both BC and biomass-burning-related organic aerosol. Overall, these results suggest that while BC and NR-PM_1_ components are influenced by common sources, their concentrations are differentially modulated by atmospheric processing and removal. Statistical differences between the three meteorological periods were evaluated using one-way ANOVA with pairwise post-hoc comparisons (Table S3). All major species and meteorological parameters showed statistically significant differences between at least two of the three periods (p < 0.001), confirming that the period classification captures genuine atmospheric regime transitions.

### Source apportionment of organic aerosols using PMF

Positive Matrix Factorization was used to derive the time series and factor profiles of the factor sources. Four factors were identified: two primary sources, such as hydrocarbon-like organic aerosol (HOA)^64,65^ and biomass-burning organic aerosol (BBOA)^66^ and two oxidized or secondary sources, named as oxygenated organic aerosol (OOA) and biomass-burning dominated OOA (BB-OOA)^67^ (Fig. 2). The time series of the four factors is displayed in Fig. 1e, while Figure S5 depicts the time series of the factors and their respective tracers. Figure S6-8 show the mass spectral profile and time series for 2, 3, and 5-factor solutions. Detailed descriptions and characteristics of each factor are mentioned below.

The Hydrocarbon-like Organic Aerosol (HOA) factor is attributed to primary emissions from fossil fuel combustion, characteristic of vehicular traffic^68^. As shown in Fig. 2d, its mass spectrum is dominated by a series of hydrocarbon-like fragments, including prominent peaks at m/z 55 (9.0%), 41 (8.3%), 57 (8.2%), 69 (7.2%), 43 (7.1%) and 71 (4.8%) (Fig. 2d). This signature pattern arises from aliphatic and cyclic hydrocarbons present in unburned fuel and lubricating oil and serves as a robust tracer for primary emissions from traffic-related combustion^68,69^. The high signals at m/z 57 and 71 are consistent with the fragments of long-chain alkyl groups typical of diesel exhaust^69^. The chemical profile is further confirmed by the very low O/C ratio of 0.08 and the high H/C ratio of 1.36, indicating a reduced, non-oxygenated chemical nature consistent with hydrocarbons. The factor profile also correlates well with reference HOA spectra (Table S2). On average, HOA contributed 11.4% to the PMF-resolved organic aerosol mass in Munnar.

The Biomass Burning Organic Aerosol (BBOA) factor is identified by its unique chemical signature originating from the combustion of cellulosic material^70,71^. The mass spectrum, shown in Fig. 2c, prominently features peaks at m/z 29 (9.6%) and 27 (4.9%), along with the critical tracer fragments at m/z 60 (4.1%) and m/z 73 (2.8%). These tracers are known fragments of anhydrous sugars, such as levoglucosan, released during the pyrolysis of cellulose and hemicellulose. Tian et al. identified the specific ions as primarily corresponding to signals at m/z 60 and m/z 73^66^. The relative intensity of m/z 60 to 73 is 1.4, which falls squarely within the characteristic range of 1.13–1.75 reported for various types of biomass burning emissions^66,72,73^. Chemically, the factor is characterized by an O/C ratio of 0.37 and an H/C ratio of 1.01, consistent with primary biomass-burning emissions. Throughout the observational campaign, BBOA constituted 13.2% of the PMF-resolved OA mass burden.

The BB-OOA factor (Fig. 2b) was identified by the presence of high *f*44 (20.9%) and *f*18 (20.9%) signals, which also have slightly higher *f*60 (0.4%) and *f*73 (0.5%), representing highly aged BB-derived aerosols. The intensity at m/z 60 was 0.004 (Fig. 2), which is greater than 0.003, which is regarded as the background level limit for biomass burning tracers^74–77^. In this case, it indicates that these secondary organic aerosols may have originated from biomass-burning emissions and undergone atmospheric aging. Previous studies from Beijing have shown that aerosols generated from biomass burning are oxidized to form low-volatility OOA^64,78^. The BB-OOA mass spectra showed a good correlation (R_UC_ = 0.7–0.93) with factor profiles of LO-OOA and SV-OOA from various other studies^75,79^. Furthermore, the *f*43 constitutes a significant fraction (7.2%), indicating a relatively less-oxidized, fresher SOA. The O/C for the factor profile was 0.98, and the H/C was 1.37, which suggests the oxidized nature of the aerosols^75^. Insufficient solar radiation, along with high RH during the monsoon, could support photochemical aqueous-phase oxidation of BBOA^67,73,75,79^. The BB-OOA factor exhibits a prominent signal (*f*91 = 1.32%) at m/z 91, suggesting a possible origin from biogenic emissions or biomass burning^80–82^. m/z 91 is also found in BBOA and HOA spectra, as it is most likely from aromatic species^80^. Previous studies have shown that non-IEPOX SOA is produced by the photo-oxidation of hydroxyl hydroperoxide (ISOPOOH) under low-NOx environments, leading to signals at m/z 91. Further studies are essential to identify the VOCs that predominate in this region during the monsoon season, contributing to SOA formation^72^. BB-OOA forms the largest fraction of the OA, according to the PMF analysis, accounting for 40.2% of the OA mass over the entire campaign.

The dominance of OOA + BB-OOA (74.5%) at this pristine high-altitude site has important implications for source attribution. OOA, with its flat diurnal profile and lack of correlation with local tracers, is consistent with a regional background source — likely aged biogenic SOA and long-range transported material arriving via the SW air mass. BB-OOA, in contrast, shows a clear diurnal cycle during the Mixed period (Section "Diurnal variations in NR-PM1 species and OA factors"), pointing to a combination of regional biomass-burning aged aerosol and locally processed emissions, as discussed in detail in Section "Source and dominance of secondary organic aerosols (SOA)". Together, these findings indicate that even at a relatively pristine high-altitude site, the submicron aerosol burden is substantially shaped by regional atmospheric processing rather than direct local emissions alone.

The OOA factor was identified by higher *f*44 (18.9%) and *f*18 (18.8%) (Fig. 2a), indicating its potential origin from secondary sources^67,73^. The OOA factor, when averaged over the entire campaign, exhibited an O/C of 0.89 and an H/C of 1.01, indicating its highly oxidized nature. This factor likely represents a regional background of aged aerosol, potentially comprising both long-range-transported material and locally formed SOA from biogenic precursors, whose temporal variability is muted by rapid mixing and wet scavenging.

The measurement site received low anthropogenic emissions due to monsoon rains and the COVID lockdown. However, the formation of OOA from the oxidation of BVOCs emitted from dense forests and tea plantations in a humid environment is highly likely, as tea gardens and forests surround the site. High relative humidity facilitates aqueous-phase and heterogeneous oxidation of organic aerosol, leading to enhanced functionalization and rapid transformation of semi-volatile organic vapors into more oxygenated organic aerosol under moist conditions, as demonstrated in laboratory and field studies of SOA oxidation at elevated RH^62,63^.

The OOA factor has a lower O/C ratio than the BB-OOA factor, as previously reported in studies showing that biomass-burning-related aerosols have higher O/C ratios than SOA from other sources^83^. Secondary biomass-burning aerosols (BB-OOA) can attain relatively high oxidation states (O/C) following atmospheric aging, occasionally reaching values comparable to or exceeding those of other oxygenated OA factors under certain conditions, as shown in chamber and field studies where aging significantly increases O/C ratios of biomass-burning OA^18,84,85^. This factor-level O/C relationship does not preclude the bulk OA being more oxidized during SW periods, when the long-range transported and highly aged OOA constitutes a larger mass fraction of total OA (Section "Source and dominance of secondary organic aerosols (SOA)").

### Influence of meteorological conditions on aerosol concentration and composition

**Fig. 4:**
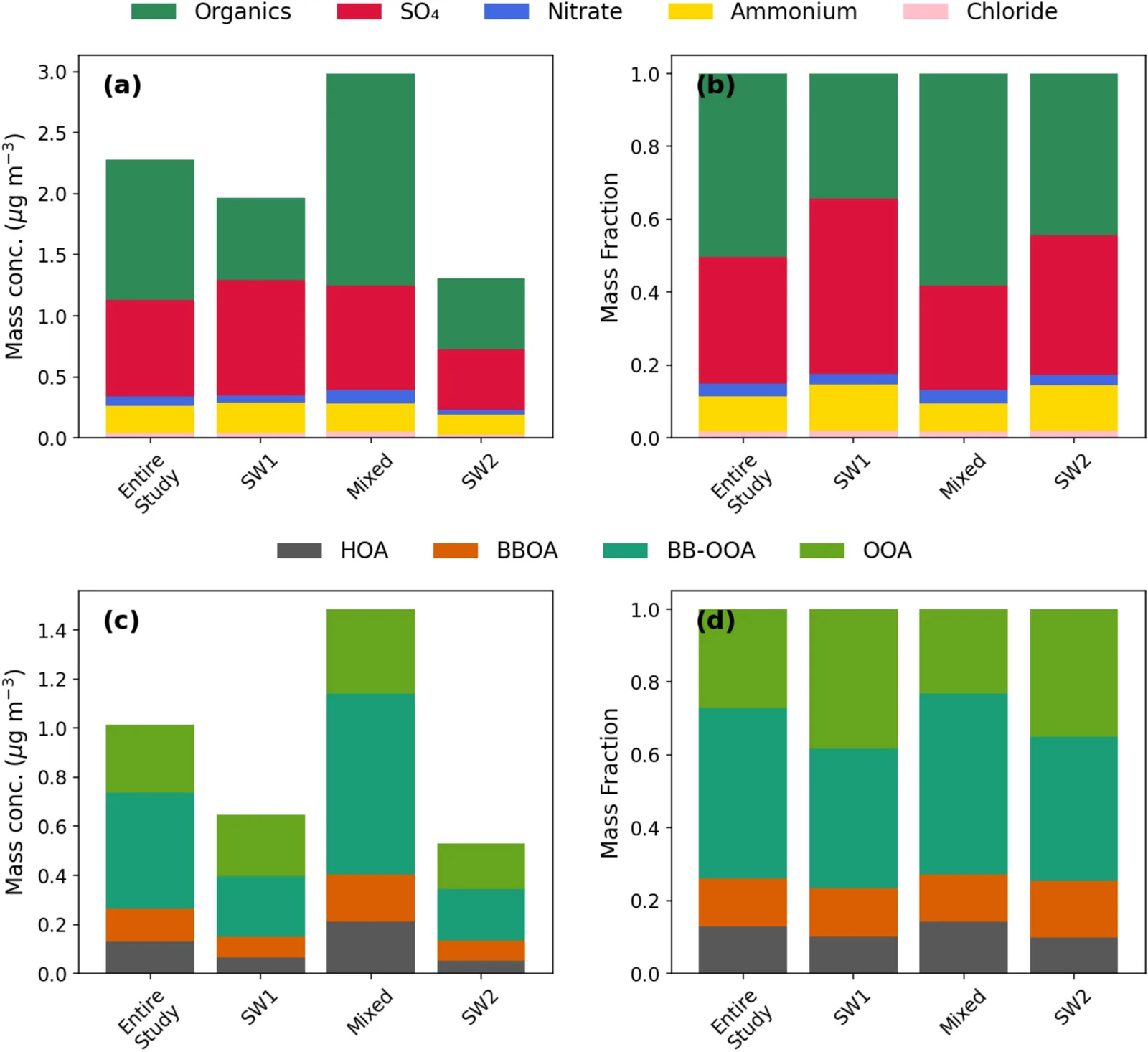
(**a**) and (**c**) Average mass concentration and (**b**) and (**d**) fraction of the measured chemical species and PMF factors during the entire campaign (06 June to 25 July), Southwest period -1 (12 June to 21 June), mixed period (22 June to 09 July), and Southwest period -2 (10 July to 25 July).

Changes in meteorological parameters played an essential role in modulating the chemical composition of the aerosols at this observational site (Fig. 1). Figure 4 shows that the total mass concentration during the SW1 and SW2 periods is less (almost half) as compared to the mixed period, indicating that the SWM disperses the aerosols due to high wind speed and also leads to the washout of aerosols due to rainfall. During the SW periods, sulfate-dominates aerosol composition, while organics reached a mass fraction of 52% in the mixed period (Figure S9). The contrasting influence of wet scavenging and transport on aerosol composition during the monsoon is consistent with observations at the high-altitude Mahabaleshwar site in the central Western Ghats, where ACSM measurements also reported reduced aerosol mass loadings during monsoon conditions under strong marine influence^42^.

The increase in organics coincided with increasing temperature above 14°C, driven primarily by BB-OOA (Figure S10a–d), consistent with enhanced photochemical processing ^23^ under the warmer conditions, characteristic of the mixed period. In contrast, the concentration of OOA remained consistently lower than that of BB-OOA and broadly unchanged, suggesting that it represents a persistent background of aged aerosol. All species and PMF factors showed decreasing concentrations at relative humidity above 90% (Figures S10e–h), likely due to wet scavenging. Higher concentrations of organics, nitrate, chloride, and PMF factors were associated with low wind speeds during the mixed period, indicating accumulation of particles from sources in the immediate vicinity. Increasing wind speeds led to a reduction in concentrations, reflecting enhanced dispersion. However, sulfate showed a different behavior, with the highest concentrations observed at wind speeds above 6 m s^-1^(Figure S10o). This pattern suggests transport from the Arabian Sea under strong W–SW winds (Figure S10k). The directional and wind-speed dependence persisted even during the Mixed period, when all organic factors shifted towards low-speed S–SE signals, whereas sulfate continued to exhibit contributions associated with stronger W–SW flow, indicating different transport characteristics for sulfate and organics (Figure S11). The relatively weak correlation between sulfate and relative humidity (R^2^ = 0.23) further argues against substantial local aqueous-phase sulfate production. While aqueous-phase processing may influence organic aerosol locally and during transport, the observed sulfate was likely associated with marine-influenced air masses and cloud processing prior to landfall rather than predominantly local formation.

The directional and wind-speed dependence of individual species and PMF factors, examined using Nonparametric Wind Regression (NWR) estimates split by meteorological period (Figures S11 and S12), provides the primary evidence for source attribution throughout this section. During the SW periods, all species show narrow signals confined to the W-SW sector (Figure S11a, c, e, g, i; Figure S12a, c, e, g), consistent with marine air masses advected from the Arabian Sea under strong monsoon flow, as confirmed by the HYSPLIT back-trajectory analysis (Figure S13). OOA shows its highest contributions at high wind speeds from the W–SW sector, indicating a predominantly long-range transported marine origin. In contrast, BB-OOA peaks at low to moderate wind speeds from the western sector, closely resembling the directional patterns of primary organic aerosol (HOA + BBOA), suggesting a stronger influence of regional biomass-burning sources^41^.

The HYSPLIT backward trajectory, residence frequency, and MODIS fire spot analyses further support these transport interpretations (Figure S13). During the SW1 and SW2 periods, air masses predominantly originated over the Arabian Sea under persistent W–SW flow and enhanced precipitation conditions, consistent with the stronger marine influence inferred from the sulfate distribution patterns. In contrast, the Mixed period exhibited slower and more regionally confined transport pathways, with greater residence time over the southern Indian landmass and enhanced regional fire activity. These conditions support the increased contribution of BBOA and BB-OOA observed during periods of low wind speed.

In contrast, the mixed-period NWR plots reveal a striking compositional and directional shift. Organics and nitrates show elevated concentrations from the S-SE sector at low wind speeds (Figure S11b, h), consistent with emissions from the densely populated Colony area in Munnar town, located in close proximity to the S-E of the measurement site. This shift is most pronounced in the primary PMF factors: HOA and BBOA both show strong S-SE low-speed signals during the mixed period (Figure S12b, d), consistent with direct traffic and residential biomass burning emissions from the Colony under the meteorologically stagnant conditions characteristic of this period (mean wind speed 2.44 ± 1.42 m s^-1^; Table S3).

BB-OOA, which was substantially higher in the mixed period, also showed enhanced low-speed signal from the S-SE sector during the mixed period (Figure S12f.), however, there are contributions from the W-SW direction as well. This wind-speed separation within the same directional sector is an important distinction: high-speed W-SW signals reflect episodic marine transport events, while the low-speed W-SW signal in BB-OOA reflects local processing of biomass-burning emissions from the tea processing facilities distributed in the vicinity of the site. Munnar houses 16 tea processing factories whose fuelwood combustion emissions, depending on prevailing wind direction, represent an important local source of primary organic aerosol that is subsequently oxidised to form BB-OOA. A recent study in a nearby town similarly attributed increases in organic content to cooking with fuelwood and open combustion of solid waste ^86^ .

OOA displays a more diffuse directional pattern, with contributions from both the E-SE and W-SW sectors. This suggests a combination of long-range transported aged aerosol and regionally formed secondary organic aerosol. Notably, the OOA mass fraction was higher during the SW periods than during the Mixed period (Figure S9), despite absolute OOA concentrations remaining broadly similar throughout the campaign, indicating that OOA represents a relatively persistent background aerosol component that is less sensitive to short-term shifts in local meteorological conditions. The campaign-average NWR distributions (Fig. 6, discussed further in Section "Source and dominance of secondary organic aerosols (SOA)") reinforce this source-speed separation across the major aerosol species and PMF factors.

### Diurnal variations in NR-PM_1_ species and OA factors

**Fig. 5:**
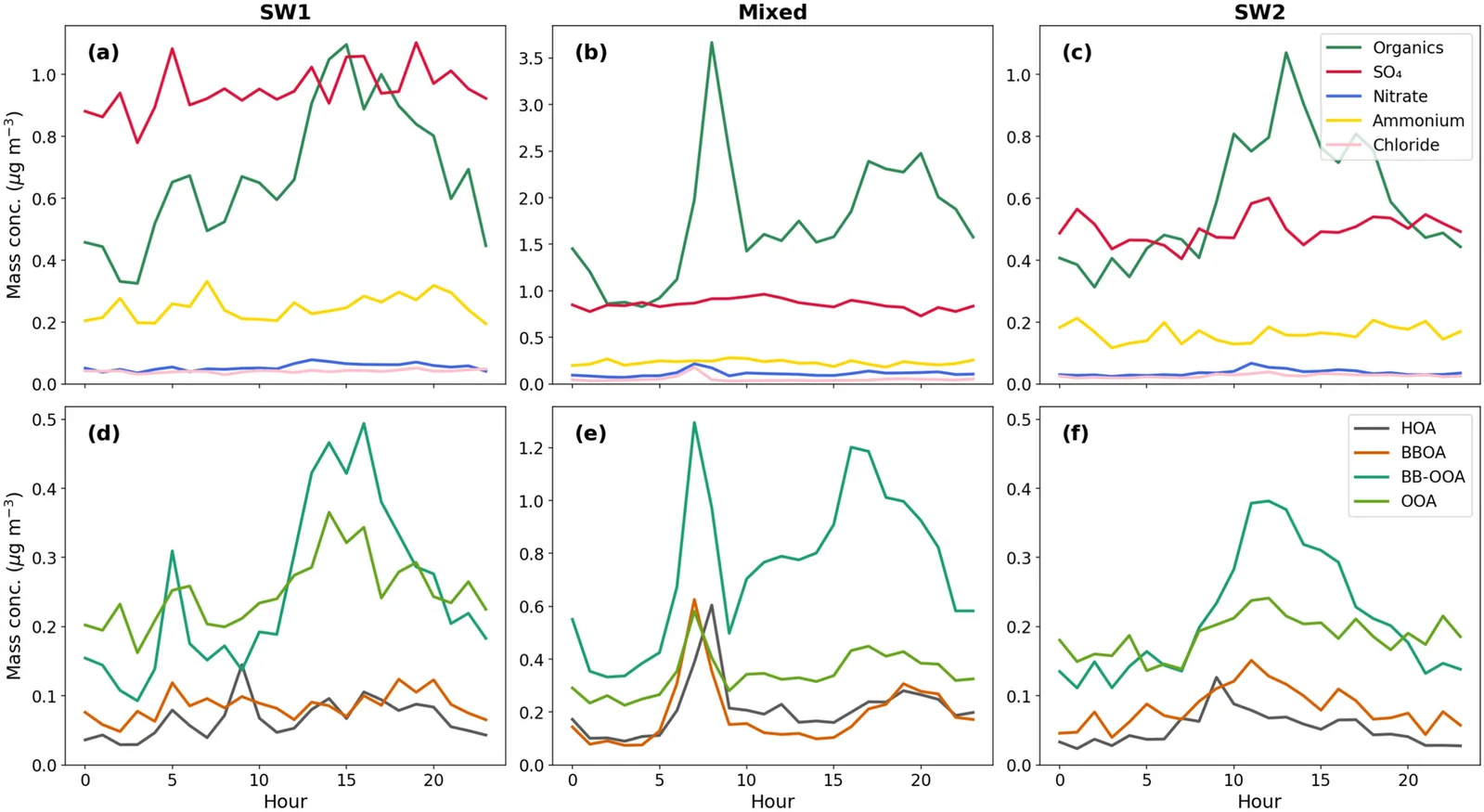
Average diurnal variations of the mass concentration of NR-PM_1_ species (Organics, SO_4_^2-^, NO_3_^-^, NH_4_^+^, and Cl^-^; top row) and the four organic aerosol factors (HOA, BBOA, BB-OOA, and OOA; bottom row) during the (**a**, **d**) SW1, (**b**, **e**) Mixed, and (**c**, **f**) SW2 periods. The figure highlights the distinct diurnal patterns between the rainy monsoon periods (SW1, SW2), which are characterized by a dominant afternoon peak in secondary organic aerosols, and the drier Mixed period, which shows a prominent morning peak from the accumulation of both primary and secondary aerosol components.

Figure 5 illustrates the diurnal patterns of non-refractory PM_1_ species and the corresponding PMF factors during the SW1, Mixed, and SW2 periods. The diel patterns of inorganic species: sulfate (SO_4_), ammonium (NH_4_^+^), nitrate (NO_3_^-^), and chloride (Cl^-^), were broadly similar across all three periods, whereas organics showed distinct variations. During the SW1 and SW2 periods, a clear daytime increase in organics was observed, suggesting dominance of photochemical oxidation of VOCs and BBOA aerosols^83^. As solar radiation intensified and the temperature reached its maximum around noon (Figure S14), an apparent increase in the secondary components, BB-OOA and OOA, was noticed (Fig. 5d and f), consistent with active photochemical oxidation^18^, while primary factors HOA and BBOA showed no corresponding increase. Continuous precipitation likely removed primary particles via wet deposition, enhancing the relative contribution of secondary aerosols to total organic mass. The period-split NWR plots confirm that BB-OOA and OOA during SW periods are associated with W-SW marine air masses at moderate-to-high wind speeds (Figure S12e, g), indicating that photochemical processing during this season occurs within marine-origin air rather than over continental source regions.

In contrast, the organics during the mixed period exhibited prominent peaks in the morning (06:00 to 10:00) and evening (18:00 to 20:00), which aligned with the diurnal cycle of traffic and residential biomass burning emissions, as reflected in the similar trend observed for the BBOA and HOA factors^66,68,85^. A detailed examination of the mixed period reveals complex aerosol processing. A sharp increase in organics and all PMF factors was observed starting at approximately 05:00. This early-morning enhancement may be attributable to the start of combustion activities, possibly from the nearby tea factory. This period was also characterized by high relative humidity (> 84%), which could facilitate the aqueous-phase processing of BBOA and HOA to form OOA and BB-OOA^63,87^. A similar but subdued peak in BB-OOA was also observed in the morning during the SW1 period. The lower levels of BB-OOA at 5 AM during the SW1 period, compared to the mixed period, could be due to rainfall washout of BBOA aerosols. As shown in Figure S10, the decrease in organics post-sunrise coincided with rising temperature and wind speed, which drive the expansion of the boundary layer and lead to the dispersion of accumulated aerosols. Whereas the OOA that shows no variation during the study, consistent with its characterization as a persistent regional background factor in previous sections.

The inorganic aerosol composition provides strong evidence for the influence of long-range transport. The concentration of sulfate remained remarkably constant with no significant diurnal variation during both the SW and Mixed periods. This lack of a daily cycle suggests it was not formed locally. It is likely that this sulfate was formed over the Arabian Sea through the oxidation of dimethyl sulfide (DMS) and was subsequently transported to the measurement site. This is supported by 72-h HYSPLIT back-trajectory analysis (Figure S13), which confirms that during all the periods, the dominant air masses originated from the Arabian Sea. The 72-h trajectory length was selected as sufficient to distinguish differences in approach pathways across periods, given the consistently marine origin of air masses throughout the campaign. During the mixed period, trajectories approach the site from a slightly NW direction and track closer to the coastline compared to SW periods, suggesting a shift in marine fetch rather than a fundamentally different air mass origin — consistent with the persistence of the high-speed W-SW sulfate signal in the NWR even during the mixed period (Figure S11d). The period from June to September is characterized by higher marine productivity in the eastern Arabian Sea due to upwelling, leading to higher expected DMS emissions. These findings are consistent with observations in Kottayam, Kerala, where secondary sulfates from biogenic marine precursors were transported inland^86^. The diurnal pattern of ammonium closely followed that of sulfate across all periods, as expected from their common association in atmospheric particles. Finally, nitrate and chloride showed a slight peak around 08:00 during the Mixed period but no diurnal variation during the SW periods, which is likely attributable to their semi-volatile nature and efficient removal by precipitation.

### Source and dominance of secondary organic aerosols (SOA)

The chemical composition and diurnal evolution of Organic Aerosol (OA) in Munnar are driven by a complex interplay among local primary emissions, boundary-layer dynamics, and regional SOA formation. When grouped into Secondary Organic Aerosol (BB-OOA + OOA) and Primary Organic Aerosol (HOA + BBOA), the secondary fraction remains the primary constituent of the organic mass across all periods (Figure S3), though its sources and formation mechanisms appear to shift with the changing meteorology.

During the active monsoon periods (SW1 and SW2), there is a slightly higher mass contribution of BB-OOA compared to the more generic OOA (Fig. 5d,f). This suggests that during the monsoon, the organic background is not merely aged regional air, but is explicitly enriched with aged biomass-burning products. The high humidity and cloud cover during these periods likely facilitate the aqueous-phase processing of biomass precursors into BB-OOA. This aligns with the elevated *f*60 values (0.014–0.015) observed in these periods (Fig. 3), confirming a persistent, processed biomass signature despite the rain.

While the SW periods show BB-OOA and OOA in closer proximity, the dry mixed period reveals a stark divergence in their concentrations (Fig. 5e). Here, BB-OOA is the overwhelming driver of total organic mass, peaking at approximately 1.3 µg m^-3^ during a sharp morning spike (07:00) and maintaining high levels with a broad evening accumulation starting around 16:00. In contrast, the OOA factor remains relatively flat and suppressed, staying below 0.4 µg m^-3^ throughout the day. The simultaneous morning peak of BB-OOA with the primary factors (BBOA and HOA) suggests that local residential and tea factory emissions are not only being released but are also either mixing with a very high background of aged biomass smoke or undergoing extremely rapid oxidation. The magnitude of the BB-OOA peak compared to OOA suggests that biomass-burning-derived precursors contribute substantially to SOA during the dry period, alongside a regional background of aged and biogenic SOA. This higher oxidation state in the SW periods likely results from the preferential washout of less-oxidized particles and the accelerated aqueous oxidation of the remaining organic fraction in the humid monsoon air.

The triangle plot prepared using *f*44 vs *f*43 (Fig. 3) is an effective tool to assess the oxidation state/atmospheric aging of OA^67^. All periods cluster near the apex of the triangle defined by *Ng *et al*. (2011)*^67^, with *f*44 values ranging from 0.17 to 0.22. These values correspond to highly oxidized aerosol, often associated with Low-Volatility OOA (LV-OOA) in previous studies. Interestingly, the SW periods exhibit higher *f*44 (~ 0.20–0.22) and calculated O:C ratios (~ 0.93–1.02) compared to the Mixed period (~ 0.17 and 0.80, respectively). This suggests that while the Mixed period has higher absolute mass, the aerosols during the monsoon are chemically more aged.

**Fig. 6:**
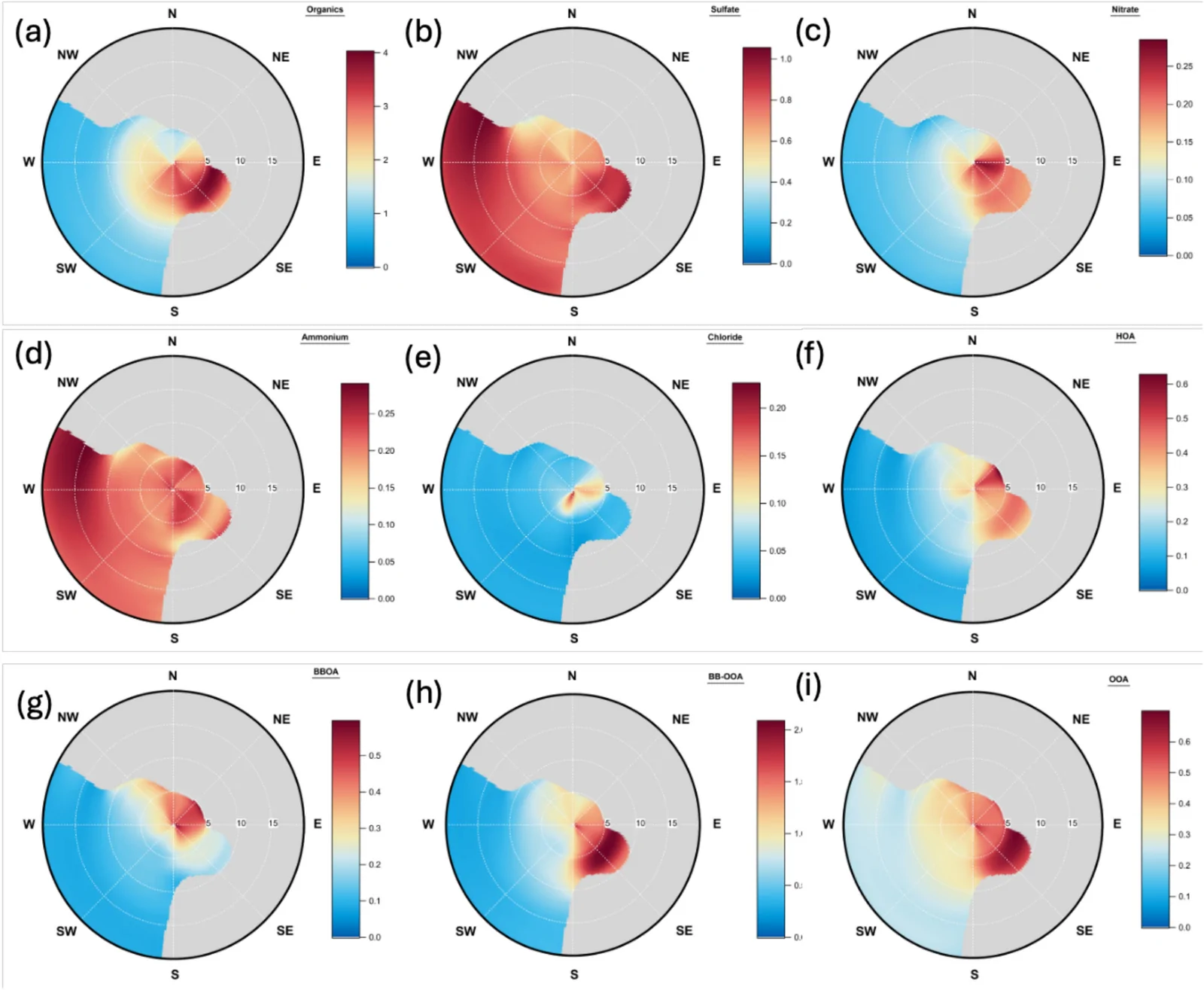
NWR estimates with the radial values representing the wind speed in m/s and color representing the mass concentration for (**a**) organics, (**b**) sulfate, (**c**) nitrate, (**d**) ammonium, (**e**) chloride, (**f**) HOA, (**g**) BBOA, (**h**) BB-OOA, and (**i**) OOA.

The campaign-average NWR estimates (Fig. 6) integrate these seasonal signals and provide a unified source-receptor picture that complements the period-split analysis in Section "Influence of meteorological conditions on aerosol concentration and composition". Biomass burning emissions originating from the southeast appear to undergo significant oxidation, forming both BB-OOA and OOA as they travel toward the site under conditions of higher relative humidity. Conversely, the highest concentrations of primary BBOA are localized in the eastern sector, towards the Munnar town. Munnar houses 16 tea processing factories; the sampling site receives fuelwood emissions from these facilities and from domestic cooking, depending on wind direction. This spatial coupling confirms that while local tea factories and residential combustion provide the primary seeds, the atmospheric processing in this high-altitude environment rapidly converts these inputs into a dominant, biomass-derived secondary aerosol layer.

## Conclusions

We conducted a study using Q-ACSM in Munnar, Kerala, in the Western Ghats, during the Southwest monsoon season from 06 June to 25 July 2021, to analyze aerosol chemical properties. Our findings show that the aerosol composition exhibits a strong dependence on meteorological regime, with organic aerosol dominating during the mixed period (52%), while sulfate accounted for a substantial fraction (43%) during the Southwest monsoon periods. Analysis of the *f*44–*f*43 triangle plot and elemental ratios indicated the predominance of aged and oxidized aerosol throughout the campaign. Enhanced sulfate contributions during the Southwest monsoon coincided with reduced PM mass loadings, reflecting the combined influence of strong monsoon winds, dilution, and wet scavenging. The dominance of sulfate and its weak association with PMF-resolved organic aerosol factors suggest a secondary origin, likely influenced by long-range transport and aqueous-phase processing in marine cloud environments prior to landfall.

PMF analysis resolved four OA factors: HOA from vehicular combustion; BBOA from local biomass burning from residential colony and 16 tea-factories in Munnar; BB-OOA representing aged biomass-burning-derived secondary aerosol; and OOA representing a highly oxidized regional background of long-range transported and biogenic secondary aerosol. Secondary factors (OOA + BB-OOA, ~ 75%) dominated organic mass across all periods, confirming that regional atmospheric processing and long-range transport exert greater control over fine aerosol composition at this site than direct local emissions. The presence of a prominent m/z 91 signal and elevated *f*60 values in the BB-OOA profile suggests rapid oxidative processing of biomass-burning emissions under high relative humidity, leading to BB-OOA formation on short timescales. The dominance of highly oxidized secondary aerosol combined with substantial sulfate contributions implies enhanced hygroscopicity, with potential implications for CCN activity and cloud microphysical processes over the Western Ghats. Future work should prioritize concurrent VOC measurements, aerosol optical property characterization, year-round Q-ACSM deployment, and size-resolved CCN measurements to further constrain the radiative and cloud-interaction implications of the aerosol burden identified here.

## Supplementary Information


Supplementary Information.


## Data Availability

The data supporting the findings of this study are archived in Figshare at 10.6084/m9.figshare.31211386.
