# Supplementary material for: Aerosol interplay with meteorology in a biodiversity-rich high-altitude site in India: impacts of southwest monsoon on aerosol chemical characteristics
Source: Sci Rep. 2026 Jun 16;16:27458. doi: 10.1038/s41598-026-57287-7 (PMC13534417; doi:10.1038/s41598-026-57287-7)
Supplement: Supplementary file 1 [file 41598_2026_57287_MOESM1_ESM.docx]

Supporting Information

Aerosol Interplay with Meteorology in a Biodiversity-Rich High-Altitude Site in India: Impacts of Southwest Monsoon on Aerosol Chemical Characteristics

Kavyashree N. Kalkura^1,2^, Aishwarya Singh^1,2,#,*^, Ramesh Chand K. A.^3^, M. Devaprasad^2^, Govindan Pandithurai^2^, Rizana Salim^1,2^, Samara Carbone^4^, James Allan^5,6^, Gordon McFiggans^5^, Pengfei Liu^7^, Hugh Coe^5^, Ravikrishna Raghunathan^2,8^, Sachin S. Gunthe^1,2,*^

^1^EE Division, Department of Civil Engineering, Indian Institute of Technology Madras, Chennai, India.

^2^Center of Atmospheric and Climate Sciences, Indian Institute of Technology Madras, Chennai, India.

^3^Department of Mechanical Engineering, College of Engineering Munnar, Munnar, India.

^4^Institute of Agrarian Sciences, Federal University of Uberlândia, Uberlândia-MG, Brazil.

^5^Department of Earth and Environmental Sciences, School of Natural Sciences, University of Manchester, Manchester, U.K.

^6^National Centre for Atmospheric Science, University of Manchester, Manchester, U.K.

^7^School of Earth and Atmospheric Sciences, Georgia Institute of Technology, Atlanta, GA, U.S.A.

^8^Department of Chemical Engineering, Indian Institute of Technology Madras, Chennai, India.

^#^Presently at the Aerosol Chemistry Department, Max-Planck Institute for Chemistry, Mainz, Germany.

***To whom correspondence should be addressed:** Aishwarya Singh ([aishwarya.singh@cacs.iitm.ac.in](mailto:aishwarya.singh@cacs.iitm.ac.in) ) and Sachin S. Gunthe ([s.gunthe@iitm.ac.in](mailto:s.gunthe@iitm.ac.in))

1. **Instrumentation**
   1.1. **Q-ACSM Tuning and Calibration**

The instrument began online real-time data collection after completing flow calibration, tuning, m/z calibration, lens alignment, calibration with ammonium nitrate and ammonium sulphate, and a zero test^1,2^. The values obtained from the calibration were applied to the measured dataset and were corrected accordingly. Flow rate coefficients calculated from the flow calibration experiment were -0.7145 (intercept) and 1.5215 (slope).

1.2. **Q-ACSM Calibration Uncertainties, QA/QC, and Reproducibility**

Calibration uncertainties: The flow calibration regression yielded a slope of 1.5215 and an intercept of −0.7145, with R² > 0.999, contributing <2% uncertainty to the volumetric flow rate. The RIE for ammonium (5.62) and sulfate (1.20) determined during mass calibration are within the typical ranges reported for Q-ACSM instruments (ammonium: 5.0-6.5; sulfate: 1.10-1.30; Crenn et al., 2015^3^), and contribute approximately ±10-15% to absolute concentration uncertainties for these species. A fixed CE of 0.5 was validated against the ion-balance method (predicted vs. measured NH₄⁺), which yielded a campaign-average ratio of 0.96 ± 0.11, indicating no systematic CE bias^4^. The combined calibration uncertainty (RIE + CE + flow) is estimated at ±30% for organics and ±25% for inorganic species, consistent with ACTRIS intercomparison estimates^2,3^.

Detection limits were determined following the methodology described in Ng et al. (2011)^1^. For 15-minute sampling, the detection limits for organics, sulphate, nitrate, ammonium, and chloride are 0.40, 0.04, 0.04, 0.33, and 0.03 µg m⁻³, respectively.

2. **PMF analysis**

Sources of submicron non-refractory organic aerosols measured in Munnar were identified through source apportionment using PMF analysis with the PET software^5^. The organic mass concentration and error matrix, calculated by the standard Q-ACSM data analysis software (acsm_local_v1.60.3), were provided as input for the analysis along with the local time. We examined a range of factors from 2 to 5 and found that a 4-factor solution presented the most meaningful solution to the dataset with a Q/Qexp = 0.21. As seen in figure 2, factor 1 represented highly aged organic aerosols with very high signals at m/z 18 and 44 and was characterized as oxygenated organic aerosols (OOA)^6^. Factor 2 represented biomass burning-influenced BB-OOA,^7^ as indicated by signals at m/z 44, 43, and 60. The mass spectrum of Factor 3 showed distinct peaks at *m/z* 60 and 73, which are established markers of fresh wood-burning emissions, and was thus classified as biomass-burning organic aerosol (BBOA)^8^. Factor 4 was characterized by dominant hydrocarbon fragment ions $({C_{n}H}_{2n+1}^{+}, \mathrm{and}$ ${C_{n}H}_{2n-1}^{+})$, consistent with hydrocarbon-like organic aerosol (HOA) and representative of primary combustion-related emissions^9,10^.

In the 3-factor solution, BBOA was not split as a separate factor profile, and the BB-OOA profile had high m/z60 signals.

In the 5-factor solution, factor 1 was most probably a split mass spectrum from various factor profiles, with its mass spectra representing no known source.

Thus, the 4-factor solution, fpeak = 0, seed = 0, was chosen as the most appropriate solution for this dataset. HOA, BBOA, OOA and BB-OOA showed similar time series, most likely due to constant POA emissions in the region throughout the campaign.

**Table S1.** Summary of PMF estimation of the Munnar dataset using the PET software

| **Factor**  **number (P)** | **Fpeak** | **Q/Q_exp_** | **Solution Description** |
| --- | --- | --- | --- |
| 2 | 0 | 0.27 | Too few factors. HOA is a mix of both BBOA and HOA. |
| 3 | 0 | 0.25 | BB-OOA and BBOA are mixed, with no separate factor profile for BBOA. |
| 4 | 0 | 0.23 | Most suitable choice of PMF factors (BBOA, HOA, OOA, and BB-OOA). Time series are correlating with the relevant tracers as shown in Figure. The first 4 factors have good correlation with standard mass spectra from AMS database. |
| 5 | 0 | 0.21 | Splitting of OOA factor was observed. |

**Table S2.** Elemental ratios and correlation of PMF factors with the reference/standard mass spectra.^11^

| **PMF factors** | **Correlation of factor profile with standard profile** | **O:C** | **H:C** |
| --- | --- | --- | --- |
| HOA | 0.87 | 0.08 | 1.36 |
| BBOA | 0.88 | 0.37 | 1.01 |
| BB-OOA | 0.87 | 0.98 | 1.37 |
| OOA | 0.67 | 0.89 | 1.01 |

**Table S3.** Statistical comparison of meteorological parameters, ACSM-derived NR-PM_1_ species, PMF factors, and black carbon (BC) across SW1, Mixed, and SW2 periods. Values are presented as mean ± one standard deviation. One-way ANOVA was used to test overall differences among the three periods, and pairwise differences were evaluated using Welch’s t-tests with Bonferroni correction. p-values < 0.05 indicate statistically significant differences.

| **Variable** | **SW1**  **(Mean ± SD)** | **Mixed (Mean ± SD)** | **SW2**  **(Mean ± SD)** | **ANOVA p-value** | **SW1 vs. Mixed** | **SW1 vs. SW2** | **Mixed vs. SW2** |
| --- | --- | --- | --- | --- | --- | --- | --- |
| **Wind Speed** | 6.45 ± 1.81 | 2.44 ± 1.42 | 6.79 ± 1.97 | 9.28E-199 | 8.12E-97 | 1.01E-01 | 8.94E-150 |
| **Temperature** | 17.07 ± 0.86 | 17.92 ± 1.76 | 16.77 ± 0.71 | 2E-34 | 1.99E-15 | 5.6E-05 | 9.97E-31 |
| **Humidity** | 96.17 ± 3.91 | 91.39 ± 6.90 | 97.74 ± 4.43 | 3.92E-54 | 1.38E-26 | 2.86E-05 | 6.6E-47 |
| **Chloride** | 0.04 ± 0.03 | 0.05 ± 0.08 | 0.03 ± 0.02 | 9.12E-32 | 4.28E-05 | 6.4E-31 | 2.25E-30 |
| **Ammonium** | 0.25 ± 0.20 | 0.23 ± 0.22 | 0.16 ± 0.18 | 4.14E-27 | 2.25E-01 | 1.14E-22 | 1.7E-20 |
| **Nitrate** | 0.05 ± 0.04 | 0.11 ± 0.10 | 0.04 ± 0.04 | 2.4E-186 | 3.09E-84 | 3.39E-23 | 2.12E-157 |
| **Sulfate** | 0.95 ± 0.32 | 0.86 ± 0.53 | 0.50 ± 0.29 | 1.07E-158 | 6.49E-08 | 7.11E-196 | 2.16E-104 |
| **Organics** | 0.67 ± 0.69 | 1.73 ± 1.68 | 0.59 ± 0.59 | 3.81E-164 | 8.97E-99 | 4.53E-03 | 3.26E-125 |
| **F1 - HOA** | 0.07 ± 0.11 | 0.21 ± 0.32 | 0.05 ± 0.10 | 2.87E-96 | 3.62E-58 | 8.99E-03 | 1.47E-74 |
| **F2- BBOA** | 0.09 ± 0.11 | 0.19 ± 0.26 | 0.08 ± 0.11 | 8.3E-65 | 1.23E-43 | 1E+00 | 6.75E-49 |
| **F3- BB-OOA** | 0.25 ± 0.29 | 0.74 ± 0.74 | 0.21 ± 0.23 | 2.25E-183 | 1.74E-110 | 5.23E-03 | 1.05E-137 |
| **F4 - OOA** | 0.25 ± 0.17 | 0.34 ± 0.25 | 0.19 ± 0.15 | 1.43E-97 | 6.7E-30 | 1.99E-18 | 9.02E-94 |
| **BC** | 0.24 ± 0.18 | 0.63 ± 1.00 | 0.16 ± 0.11 | 8.67E-88 | 6.06E-48 | 6.43E-31 | 3.4E-69 |

**Figure S1.** Summary of the key diagnostic plots of the chosen 4-factor solution for the Munnar ACSM data, from PMF analysis using PET software.

**Figure S2.** Daily precipitation as measured using a tipping-bucket rain gauge (Campbell Scientific TB4) operated by the National Centre for Earth Science Studies (NCESS) at the Cloud Physics Observatory in Munnar, located approximately 10 km from the study site.

**Figure S3.** The diurnal variation of mean mass concentration of SOA (OOA + BB-OOA) and POA (HOA + BBOA) during a) First Southwest period (SW1) b) Mixed period and c) Second Southwest period (SW2).

**Figure S4.** Correlation plots between BC (averaged on ACSM time resolution) and the four PMF factors.

**Figure S5.** Time series for the four-factor solution of PMF analysis, along with the time series of its respective tracers.

**Figure S6.** Mass spectral profile and time series for 2-factor solution (fpeak =0) of PMF analysis.

**Figure S7.** Mass spectral profile and time series for 3-factor solution (fpeak =0) of PMF analysis.

**Figure S8.** Mass spectral profile and time series for 5-factor solution (fpeak =0) of PMF analysis.

**Figure S9.** Average chemical composition of NR-PM_1_ and PMF factors during the (a, d) SW1, (b, e) Mixed, and (c, f) SW2 periods. The top row shows the mean mass fractions of ACSM species, and the bottom row shows the corresponding contributions of PMF factors.

**Figure S10.** Mean mass concentration and fraction of aerosol species as a function of (a-d) temperature, (ºC) (e-h) relative humidity (%), (i-k) wind speed (m/s), and (m-p) wind direction.

**Figure S11.** NWR estimates for the NR-PM_1_ species measured by ACSM during SW periods (left) and mixed period (right), with the radial values representing the wind speed in m/s and colour representing the mass concentration.

**Figure S12.** NWR estimates for the PMF-derived OA factors during SW periods (left) and mixed period (right), with the radial values representing the wind speed in m/s and colour representing the mass concentration.

**Figure S13.** Air mass back trajectories, residence frequency distributions, and MODIS fire activity during the three meteorological regimes identified during the campaign: (a, d, g) SW1 (12–21 June), (b, e, h) Mixed (22 June–09 July), and (c, f, i) SW2 (10–25 July). Panels (a–c) show 72-hour HYSPLIT backward trajectories arriving at the study site (black triangle) at 250 m above ground level, computed using 1° x 1° GDAS meteorological data. The shaded background represents mean daily precipitation (mm day⁻¹) derived from ERA5 reanalysis data. Panels (d–f) show the normalized residence frequency distributions corresponding to the trajectory ensembles, while panels (g–i) show MODIS fire spots colored by fire radiative power (MW). The SW1 and SW2 periods were characterized by persistent transport from the Arabian Sea under strong W–SW flow and enhanced precipitation, whereas the Mixed period exhibited slower and more regionally confined transport pathways together with enhanced regional fire activity.

(a)

(b)

**Figure S14.** The diurnal variation of mean temperature, relative humidity and wind speed during a) the SW periods (SW1 and SW2) and b) the Mixed period.

References:

1.	Ng, N. L. *et al.* An Aerosol Chemical Speciation Monitor (ACSM) for routine monitoring of the composition and mass concentrations of ambient aerosol. *Aerosol Science and Technology* **45**, 780–794 (2011).

2.	Freney, E. *et al.* The second ACTRIS inter-comparison (2016) for Aerosol Chemical Speciation Monitors (ACSM): Calibration protocols and instrument performance evaluations. *Aerosol Science and Technology* **53**, 830–842 (2019).

3.	Crenn, V. *et al.* ACTRIS ACSM intercomparison - Part 1: Reproducibility of concentration and fragment results from 13 individual Quadrupole Aerosol Chemical Speciation Monitors (Q-ACSM) and consistency with co-located instruments. *Atmos. Meas. Tech.* **8**, 5063–5087 (2015).

4.	Canagaratna, M. R. *et al.* Chemical and microphysical characterization of ambient aerosols with the aerodyne aerosol mass spectrometer. *Mass Spectrom. Rev.* **26**, 185–222 (2007).

5.	Ulbrich, I. M., Canagaratna, M. R., Zhang, Q., Worsnop, D. R. & Jimenez, J. L. Interpretation of organic components from Positive Matrix Factorization of aerosol mass spectrometric data. *Atmos. Chem. Phys.* **9**, 2891–2918 (2009).

6.	Zhang, Q., Worsnop, D. R., Canagaratna, M. R. & Jimenez, J. L. *Hydrocarbon-like and Oxygenated Organic Aerosols in Pittsburgh: Insights into Sources and Processes of Organic Aerosols*. *Atmos. Chem. Phys* vol. 5 www.atmos-chem-phys.org/acp/5/3289/SRef-ID:1680-7324/acp/2005-5-3289EuropeanGeosciencesUnion (2005).

7.	Kommula, S. *et al.* Chemical Characterization and Source Apportionment of Organic Aerosols in the Coastal City of Chennai, India: Impact of Marine Air Masses on Aerosol Chemical Composition and Potential for Secondary Organic Aerosol Formation. *ACS Earth Space Chem.* **5**, 3197–3209 (2021).

8.	Ng, N. L. *et al.* Organic aerosol components observed in Northern Hemispheric datasets from Aerosol Mass Spectrometry. *Atmos. Chem. Phys.* **10**, 4625–4641 (2010).

9.	Sun, Y. L. *et al.* Aerosol composition, sources and processes during wintertime in Beijing, China. *Atmos. Chem. Phys.* **13**, 4577–4592 (2013).

10.	Zhang, J. K. *et al.* Characterization of submicron aerosols during a month of serious pollution in Beijing, 2013. *Atmos. Chem. Phys.* **14**, 2887–2903 (2014).

11.	Ng, N. L. *et al.* Real-time methods for estimating organic component mass concentrations from aerosol mass spectrometer data. *Environ. Sci. Technol.* **45**, 910–916 (2011).
